# Interleukin 20 receptor A expression in colorectal cancer and its clinical significance

**DOI:** 10.7717/peerj.12467

**Published:** 2021-11-16

**Authors:** Rui Liu, Honghao Yin, Xin Sun, Songyi Liu, Ang Wang, Ying Wu, Yuan Yuan, Yuehua Gong, Chengzhong Xing

**Affiliations:** 1 Key Laboratory of Cancer Etiology and Prevention in Liaoning Education Department, the First Hospital of China Medical University, Shenyang, China; 2 Tumor Etiology and Screening Department of Cancer Institute and General Surgery, the First Hospital of China Medical University, Shenyang, China; 3 Key Laboratory of GI Cancer Etiology and Prevention in Liaoning Province, the First Hospital of China Medical University, Shenyang, China

**Keywords:** Colorectal cancer, IL20RA, IHC, Differentially expressed genes, Regulatory network

## Abstract

**Background:**

Interleukin 20 receptor A (IL20RA) has been shown to play a role in the establishment and progression of multiple tumors. However, the expression of this protein in colorectal cancer (CRC) and its correlation with the clinicopathological parameters of CRC have remained unclear.

**Methods:**

A total of 323 paraffin sections including CRC tissues and adjacent normal tissues after surgery were collected. IL20RA protein expression was detected by immunohistochemical staining. The difference expression of IL20RA mRNA between CRC and normal tissues was also explored in the Oncomine and GEO databases. In addition, the IL20RA-related differentially expressed genes were analyzed in TCGA database and enrichment analysis was conducted to explore the cell functions and pathways related to IL20RA expression.

**Results:**

There was increased IL20RA expression in CRC compared with that in normal tissues. High IL20RA expression was associated with greater tumor diameter, lymph node metastasis, and poor TNM stage in CRC, while also being suggestive of poor prognosis. The main pathways of IL20RA-related differentially expressed genes in TCGA were protein heterodimerization activity, oxygen binding, oxygen transporter activity, hormone activity, and lipid transporter activity. Meanwhile, IL20RA-related differentially expressed genes were mainly enriched in peroxidase, nucleotide stimulant repair, fatty acid metabolism, basal transcription factor, and RNA degradation.

**Conclusions:**

IL20RA might have a role as a biomarker for CRC. Its upregulation might contribute to an aggressive phenotype in CRC. IL20RA’s involvement in the development and progression of CRC might occur through it affecting fatty acid metabolism, oxygen binding, oxygen transport, and hormone activity.

## Introduction

Colorectal cancer (CRC), one of the most common malignant tumors, is associated with high morbidity and mortality (Baisse et al., 2001; Ursic-Vrscaj et al., 1994). According to the latest cancer statistics, in China CRC accounted for one-fifth of all malignant tumors, with 376,000 new cases and 191,000 deaths (Chen et al., 2016). Precise prevention early diagnosis, and standardized treatment are effective ways to reduce the morbidity and mortality of CRC, which are strongly dependent on a deep understanding of the characteristics of this disease. Under this background, it is essential to explore the key events as well as biomarkers involved in the CRC progression.

Interleukins (ILs) are cytokines produced by many cells that in turn act on many cells. They play important roles in transmitting information, activating and regulating immune cells, mediating T- and B-cell activation, proliferation, and differentiation, and inflammatory responses. Recently, ILs and their receptors were reported to be involved in tumor progression (Yamazumi et al., 2006). IL20 is a newly discovered cytokine assigned to the interleukin 10 families, which is produced by activated peripheral blood mononuclear cells and keratinocytes (Rätsep et al., 2008). IL20R had two subunits, IL20RA and IL20RB, both of which are orphan receptors of the type II receptor family. Normally, IL20R is expressed in a variety of immune cells, especially Th2 cells. It can also be expressed in normal tissues such as keratinocytes (Sa et al., 2007), airway epithelial cells (Huang et al., 2008), and other endothelial cells (Jain et al., 2011). The role of IL20R in tumors has also recently been revealed. Swarts et al. (2013) reported that IL20RA was downregulated in lung carcinoid tumors and could be used as an independent prognostic factor for such tumors. Wigle et al. (2002) also showed that the low expression of IL20RA caused by hypermethylation of DNA CpG islands was associated with poor disease-free survival of non-small cell lung cancer. However, whether IL20RA could serve as a new biomarker has remained unclear. Moreover, the protein expression of IL20RA in CRC and its relationship with clinicopathological parameters have yet to be resolved.

In this study, we investigated the expression of IL20RA in CRC and analyzed its relationship with the clinicopathological parameters and prognosis of CRC, aimed to clarify its potential as a predictive and prognostic marker of CRC. Furthermore, we explored the genes particularly associated with IL20RA expression and the expression-related interaction networks by bioinformatic analysis.

## Materials and Methods

### Patients and tissue specimens

Postoperative paraffin specimens from CRC patients who underwent radical resection at the Anorectal Surgery Unit of China Medical University from November 2012 to September 2017 were collected. These included 155 CRC and 168 adjacent normal cases. All pathological diagnoses were in accordance with the 2010 World Health Organization (WHO) Classification of Tumors of the Digestive System. The patients were followed up by telephone for 6 months after the operation to collect data on postoperative recurrence, metastasis, and survival. Additionally, we collected detailed clinical information, including gross classification, histological type, growth pattern, depth of invasion, peripheral nerve invasion, vascular tumor thrombus, lymph node metastasis, extranodal tumor implantation (ENTD), and staging of the tumor-lymph node-metastasis (TNM) system. This study was approved by the Ethics Committee of the First Affiliated Hospital of China Medical University and written informed consent was obtained from all enrolled patients.

### Immunohistochemical (IHC) staining

IHC staining was performed with reference to a previous report (Deng et al., 2014). Briefly, 4-µm-thick, formalin-fixed, paraffin-embedded sections were cut, routinely deparaffinized, rehydrated, and heated in antigen retrieval citrate buffer (pH 6.0). Subsequently, endogenous peroxidase was blocked using 3% hydrogen peroxide for 20 min and non-specific antigen was blocked with 10% normal goat serum at 37 °C for 20 min. Then, the rabbit polyclonal antibody anti-IL20RA (TA349232; 1:300 dilution; Origene, Maryland, USA), Anti-Ki67 (MAB-0672; MXB, China), Anti-p53 (MAB-0674; MXB, China), Anti-EGFR (RMA-0804; MXB, China) was incubated for 1 h at room temperature, followed by conjugation to the secondary antibody (goat anti-rabbit antibody; Maixin Inc., Fujian, China) and DAB (C-0010) staining. Primary antibody replaced with PBS buffer was used as a negative control.

### Evaluation of IHC staining

The IHC results were evaluated and scored independently by two investigators who were blinded to the patients’ clinicopathological characteristics. Protein expression was evaluated using a semi-quantitative scoring system based on the intensity scores (InS) (0, no staining; 1, light brown staining; 2, brown staining; and 3, dark brown staining) and proportion scores (PS) of stained epithelial cells (0%–5% (0), 6%–25% (1), 26%–50% (2), 51%–75% (3), and 76%–100%). The final immunohistochemical scores (IS) were calculated using the following formula: IS = PS × InS. Finally, the IL20RA expression was classified into four grades as follows, in accordance with a previous report (Feng et al., 2018): negative (–), score = 0; weak expression (+), score = 0.5–4; moderate expression (++), score = 4.5–8; and strong expression (+++), score = 9–12.

### Oncomine and Gene Expression Omnibus (GEO) analyses

The public Oncomine database and GEO dataset (accession number GSE10950), which contains 24 pairs of normal colon and tumor tissues using Illumina BeadChip Human Ref8-v2, were used to verify IL20RA expression in CRC. And *P*-value < 0.01 and log fold change (logFC) > 1.5 were regarded as statistically significant.

### Screening for IL20RA-related DEGs in TCGA database

The Cancer Genome Atlas (TCGA) database (https://portal.gdc.cancer.gov/) was used to screen IL20RA-related DEGs. A total of 488 CRC patients were divided into two groups according to the median value of IL20RA expression. R package (edgeR) was used to analyze the genes that were differentially expressed between the two groups (Robinson, McCarthy & Smyth, 2010). False discovery rate (FDR) <0.05 and —logFC— > 1.5 were regarded as significant. Our research complied with the publication guidelines provided by TCGA (http://cancergenome.nih.gov/publications/publicationguidelines).

### Pathway enrichment analysis of IL20RA-related DEGs

Kyoto Encyclopedia of Genes and Genomes (KEGG), a database containing information on genomes, biological pathways, diseases, and chemicals was used for understanding high-level functions and utilities of the biological system, such as the cell, the organism, and the ecosystem, from molecular-level information, especially large-scale molecular datasets generated by genome sequencing and other high-throughput experimental technologies.

### Protein–protein interaction network (PPI) related to IL20RA expression

PPI network was generated using the STRING database (Szklarczyk et al., 2019). The protein regulatory network was visualized and analyzed with Cytoscape 3.4.0 (Otasek et al., 2019). We used Centiscape 2.2 to calculate the degree of centrality and defined the hub genes associated with IL20RA expression based on degree ≥14 (Scardoni, Petterlini & Laudanna, 2009). MOCE’s k-core analysis was used to help screen out stable structures in the network and narrow the scope of research.

### Statistical analysis

SPSS 20.0 was used for statistical analysis. A non-parametric test was used to detect the difference in IL20RA protein expression between CRC and normal tissues. The receiver operating characteristic (ROC) curve was established to evaluate the diagnostic value of IL20RA for CRC. The area under the curve(AUC) between 0.70 and 0.90 was considered to be of high diagnostic value (Johann Jr et al., 2004). Pearson Correlation Coefficient(PCC) was used to analyze the expression correlation between IL20A and several immunohistochemical staining markers in CRC. Fisher’s exact test was used to analyze the correlation of IL20RA expression with clinicopathological parameters. X-tile software was used to explore the best cut-off value of the IHC score, according to which CRC patients were divided into high- and low-risk groups, as well as high- and low-IL20RA-expression groups. The relationship between IL20RA and CRC survival was evaluated using a multivariate Cox proportional hazards model. *P* < 0.05 was regarded as significant.

## Results

### Baseline characteristics of the subjects

A total of 323 postoperative paraffin specimens from CRC patients were included in this study, namely, 155 CRC tissues and 168 adjacent normal tissues. The subjects included 177 males and 146 females; 140 were aged <60 and 183 were ≥60 years old, with a mean age of 60.59 ± 11.80 years old. The 155 cases of CRC included 47 cases of colon cancer and 108 cases of rectal cancer.

### IL20RA was upregulated in CRC tissues compared with that in normal and can be used as a diagnostic biomarker

**Figure 1:**
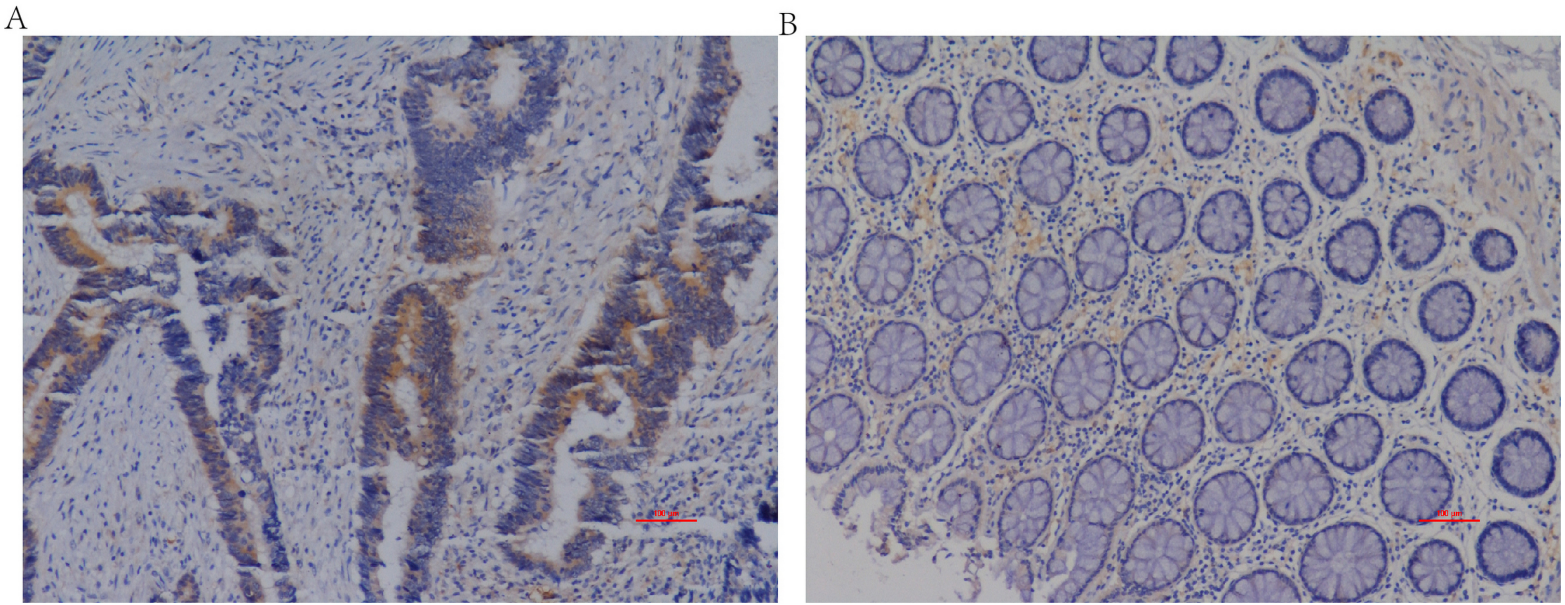
Representative IL20RA protein expression in CRC and adjacent normal tissues. (A) CRC tissue, (B) adjacent normal tissue. Magnification, ×100.

**Figure 2:**
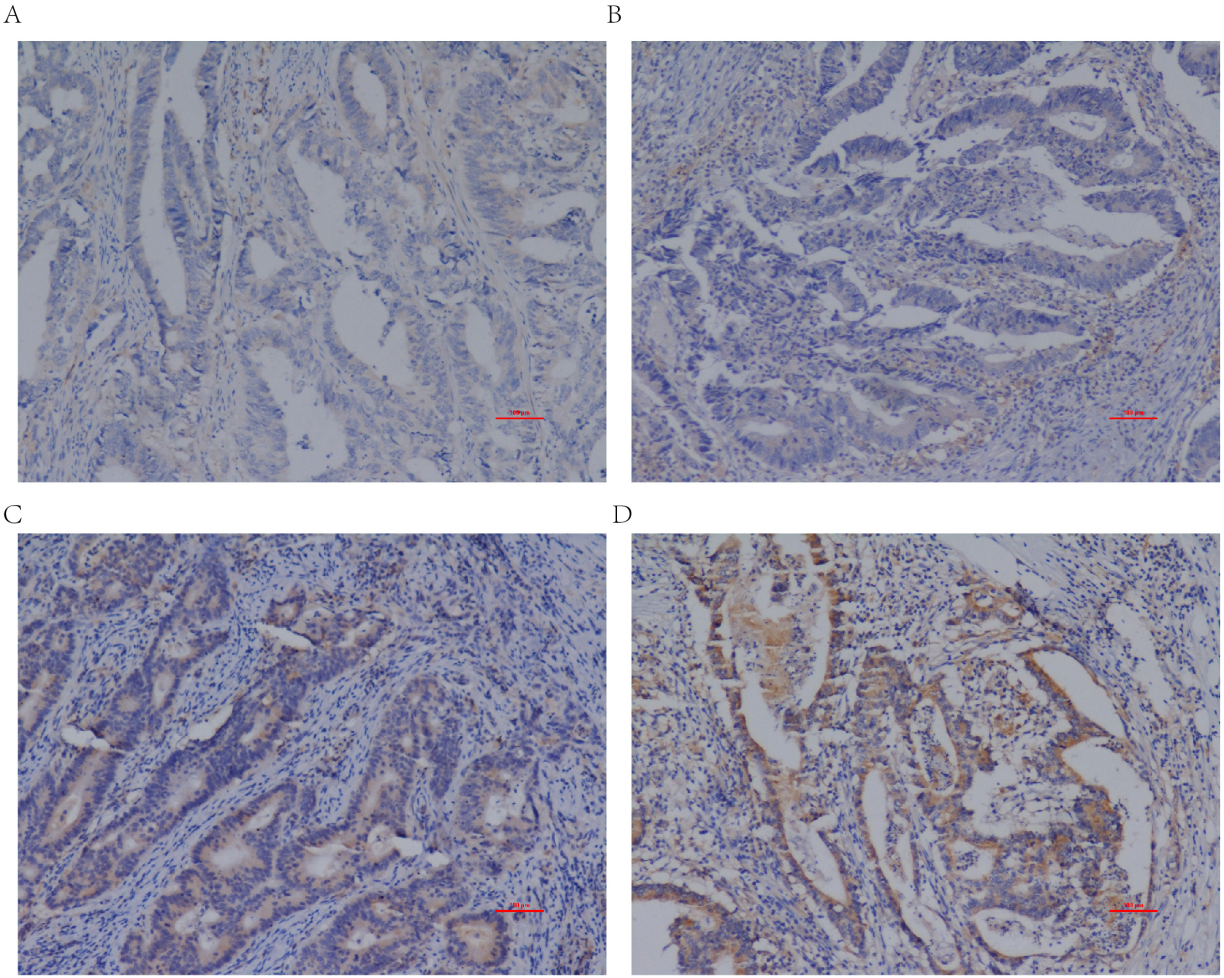
Differential IL20RA expression levels in CRC tissues. (A) negative (-), (B) weakly positive (+), (C) moderately positive (++), and (D) strongly positive (+++). Magnification, ×100.

**Figure 3:**
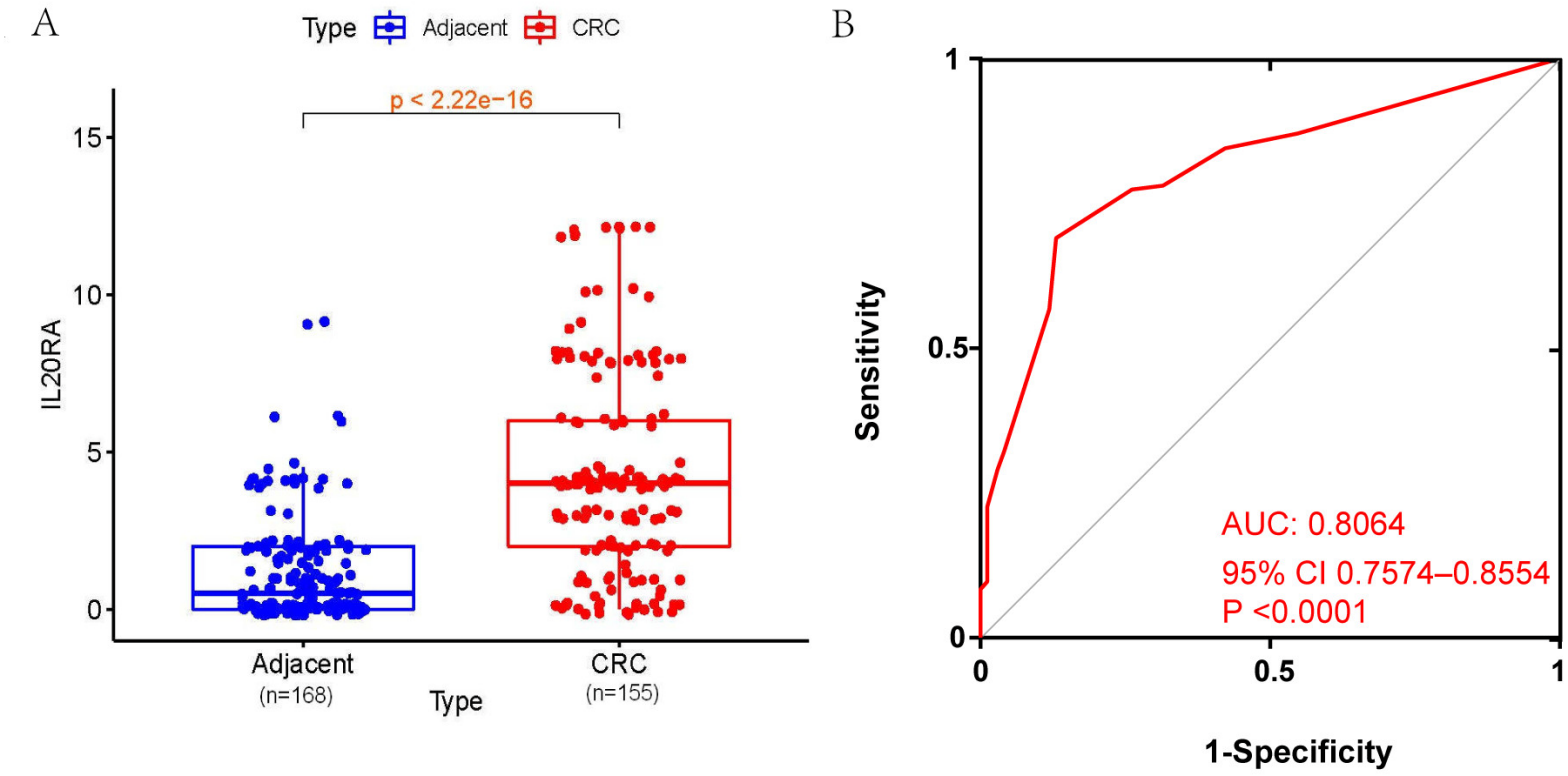
IL20RA protein expression in CRC tissues compares with adjacent normal tissues and its diagnostic value. (A) IL20RA protein expression in CRC tissues compares with adjacent normal tissues, (B) ROC curves of IL20RA.

The IHC staining of CRC and adjacent normal tissues was shown in Fig. 1. Figure 2 presented four different grades of IL20RA protein expression in CRC: negative (−), weakly positive (+), moderately positive (++), and strongly positive (+++). By the non-parametric test, we found IL20RA protein expression in CRC tissue was significantly increased compared with that in normal tissues (*P* < 0.01), as shown by the box plot in Fig. 3A. Subgroup analysis according to age, sex, and tumor location indicated consistently significant upregulation of IL20RA protein in CRC tissues compared with that in adjacent tissue. ROC result indicated IL20RA (Area = 0.8064, 95% CI [0.7574–0.8554], and *P* < 0.0001) was a good predictor for CRC prognosis (Fig. 3B).

### The expression correlation between IL20RA and Ki-67, P53, EGFR

Among all CRC tissue samples used in this study, 120 cases were selected for expression correlation analysis which contained the IHC staining results of Ki-67, P53, and EGFR. The expression results of all markers scored as described in *Methods*. PCC showed no significant expression correlation between IL20RA and Ki-67 (*R* = 0.004, *P* = 0.967), P53(R =−0.037, *P* = 0.685), EGFR(*R* = 0.027, *P* = 0.774) in Table S1.

**Table 1:** The differential expression of IL20RA protein between CRC and adjacent normal tissues.

**Parameters**	**Tissues(n)**	**+++**	**++**	**+**	**-**	***P-value***
		**n**	**n**	**n**	**n**	
Overall	CRC(155)	15(9.7)	35(22.6)	85(54.8)	20(12.9)	**0.0001**
	Normal(168)	2(1.2)	5(3.0)	85(50.6)	76(45.2)	
Male	CRC(85)	11(12.9)	18(21.3)	45(52.9)	11(12.9)	**0.0001**
	Normal(92)	2(2.2)	3(3.2)	47(51.1)	40(43.5)	
Female	CRC(70)	4(5.7)	17(24.3)	40(57.1)	9(12.9)	**0.0001**
	Normal(76)	0(0.0)	2(2.6)	38(50.0)	36(47.4)	
<60	CRC(67)	6(9.0)	12(17.9)	39((58.2)	10(14.9)	**0.0001**
	Normal(73)	2(2.7)	2(2.7)	32(43.8)	37(50.8)	
≥60	CRC(88)	9(10.2)	23(26.1)	46(52.3)	10(11.4)	**0.0001**
	Normal(95)	0(0.0)	3(3.1)	53(55.8)	39(41.1)	
Colon	CRC(47)	8(17.1)	9(19.1)	25(53.2)	5(10.6)	**0.0001**
	Normal(41)	1(2.4)	1(2.4)	22(53.7)	17(41.5)	
Rectum	CRC(108)	7(6.5)	26(24.1)	60(55.6)	15(13.8)	**0.0001**
	Normal(127)	1(0.8)	4(3.1)	63(49.6)	59(46.5)	

### Relationship between IL20RA protein expression and clinicopathological parameters of CRC patients

To evaluate the relationship between IL20RA protein expression and clinicopathological parameters, subgroup analysis was carried out according to variables including age, sex, tumor site, depth of invasion, lymph node metastasis, TNM staging, tumor growth pattern, tissue grading, vascular tumor thrombus, extracolonic implantation, tumor size, ganglion invasion, pericancerous lymphatic infiltration, and histological classification (Table 1). The results indicated that CRC patients with tumor size >4 cm had higher IL20RA expression than those with <4 cm (*P* = 0.023). CRC patients with lymph node metastasis had significantly higher IL20RA expression than those without it (*P* = 0.017). In addition, CRC patients in T3/T4 stage exhibited the overexpression of IL20RA compared with those in T1/T2 stage (*P* = 0.023). However, the protein expression of IL20RA in CRC was not significantly associated with other clinicopathological parameters (*P* > 0.05) (Table 2).

**Table 2:** The correlation between IL20RA protein expression and clinicopathological parameters of CRC.

**Parameters**	**Expression level**				***P*** **-value**
	**+++**	**++**	**+**	**-**	
	**n(%)**	**n(%)**	**n(%)**	**n(%)**	
Age					0.61
<60	6(8.96)	12(17.91)	39(58.2)	10(14.93)	
≥60	9(10.2)	23(26.1)	46(52.3)	10(11.4)	
Gender					0.501
Male	11(12.9)	18(21.2)	45(52.9)	11(13.0)	
Female	4(5.7)	17(24.3)	40(57.1)	9(12.9);	
Invasion Depth					0.23
T1+T2	2(3.5)	14(24.6)	32(56.1)	9(15.8)	
T3+T4	13(13.3)	21(21.4)	53(54.1)	11(11.2)	
Lymph node metastasis					**0.017**
Negtive	4(4.1)	23(23.5)	56(57.1)	15(15.3)	
Positive	11(19.3)	12(21.1)	29(50.9)	5(8.7)	
TNM Stage					**0.023**
I–II stage	4(4.2)	23(24.2)	53(55.8)	15(15.8)	
III–IV stage	11(18.3)	12(20.0)	32(53.3)	5(8.4)	
Tumor location					0.22
Colon	8(17.0)	9(19.1)	25(53.2)	5(10.7)	
Rectum	7(6.5)	26(24.1)	60(55.6)	15(13.9)	
Growth pattern					0.484
Ulcerative	13(11.4)	27(23.7)	61(53.5)	13(11.4)	
Others	2(4.9)	8(19.5)	24(58.5)	7(17.1)	
Differentiation degree					0.489
Well	8(7.3)	26(23.9)	61(56.0)	14(12.8)	
Poor	7(15.2)	9(19.6)	24(52.2)	6(13.0)	
Vascular cancer thrombus					0.63
Negative	10(9.2)	29(26.6)	59(54.1)	11(10.1)	
Positive	5(14.3)	6(17.1)	20(57.1)	4(11.5)	
Tumor deposits					0.094
Negative	5(25.0)	3(15.0)	11(55.0)	1(5.0)	
Positive	8(7.8)	28(27.2)	55(53.4)	12(11.7)	
Maximum diameter (cm)					**0.023**
≦4cm	1(1.7)	14(23.3)	39(65.0)	6(10.0)	
>4cm	14(16.7)	21(25.0)	40(47.6)	9(10.7)	
Ganglion invasion					0.295
Negative	12(14.3)	20(23.8)	43(51.2)	9(10.7)	
Positive	1(2.7)	11(29.7)	21(56.8)	4(10.8)	
Lymphatic invasion					0.678
Negative	2(11.1)	2(16.7)	12(66.7)	1(5.6)	
Positive	13(11.0)	30(25.4)	62(52.5)	13(11.1)	
Tissue type					0.347
Adenocarcinoma	12(8.6)	33(23.6)	78(55.7)	17(12.1)	
Mucinous adenocarcinoma	3(20.0)	2(13.3)	7(46.7)	3(20.0)	

### High IL20RA protein expression in CRC was associated with poor prognosis

**Figure 4:**
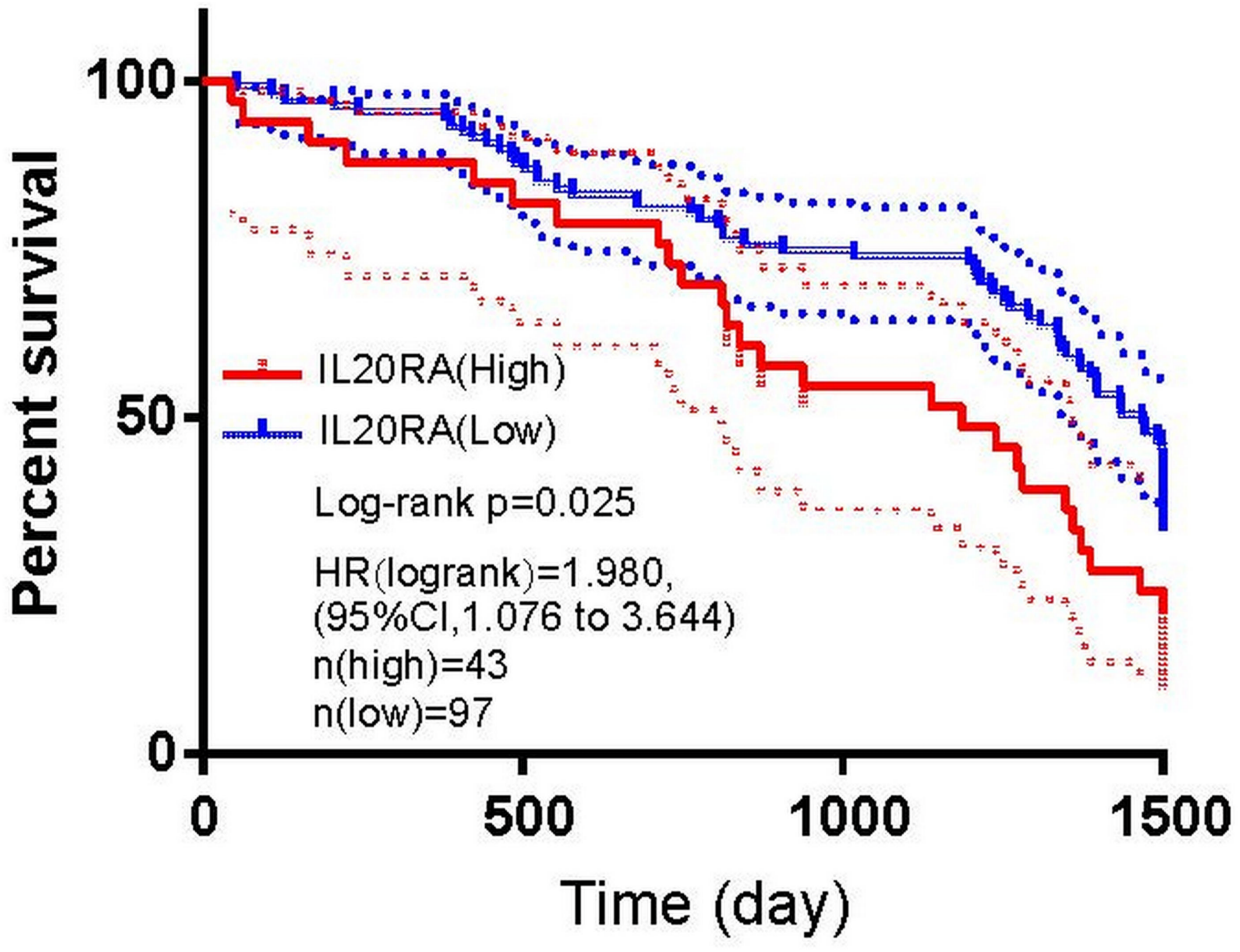
The prognostic value of IL20RA protein expression in CRC. High IL20RA protein expression predicted poor prognosis.

To evaluate the association between the expression of IL20RA and the survival of CRC, the Cox proportional hazards model was adopted. By X-tile analysis, the IHC score of 6 was selected as the cut-off (IHC score > 6 means high IL20RA expression, and ≤ 6 means low IL20RA expression). Univariate Cox proportional hazards model indicated that the higher IL20RA expression, the shorter the survival (hazard ratio (HR) = 1.980, 95% confidence interval (CI): 1.076–3.644, *P* = 0.025; Fig. 4). Multivariate Cox regression analysis adjusted by age, sex, tumor site, tumor size, tumor differentiation degree, and TNM stage, showed that IL20RA protein expression (HR = 1.975, 95% CI [1.068–3.653], *P* = 0.030), the degree of tumor differentiation (HR = 2.013, 95% CI [1.110–3.651], *P* = 0.021), and TNM stage (HR = 1.823, 95% CI [1.006–3.305], *P* = 0.048) had an impact on the survival of CRC patients (Table 3).

**Table 3:** Univariate and multivariate analysis of survival in CRC patients.

**Variables**	**Cases**	Univariable Analysis		Multivariable Analysis	
		Hazard Ratio (95% CI)	*P* value	Hazard Ratio (95% CI)	*P* value
IL20RA expression (High *vs.* Low)	140	1.980 (1.076–3.644)	0.025	1.975 (1.068–1.975)	**0.030**
Differentiation (Low *vs.*. high)	140	2.162 (1.209–3.866)	0.008	2.013 (1.110–3.651)	**0.021**
TNM stage (III+IV *vs.*. I+II)	140	2.116 (1.181–3.7920)	0.010	1.823 (1.006–3.305)	**0.048**
Sex (male *vs.* female)	140	1.977 (1.055–3.708)	0.030	1.590 (0.834–3.033)	0.131
Lymph node metastasis (Yes *vs.*. No)	140	2.001 (1.121–3.573)	0.017	1.827 (0.378–8.829)	0.372
Tumor size (>4 *vs.*≤4)	139	1.885 (0.989–3.594)	0.049	1.223 (0.608–2.462)	0.098

### IL20RA mRNA expression in Oncomine and GEO databases

IL20RA mRNA expression was found to be upregulated in CRC compared with the level in normal tissue in the Oncomine (IL20RA in Skrzypczak colorectal dataset, fold change = 2.034, *P* value = 1.63E −16) and GEO databases (IL20RA in GSE10950 colorectal dataset, fold change = 1.996, FDR = 1.88E −14), which was consistent with our results on IL20RA protein expression in CRC.

### IL20RA-related DEGs obtained from the TCGA database

**Figure 5:**
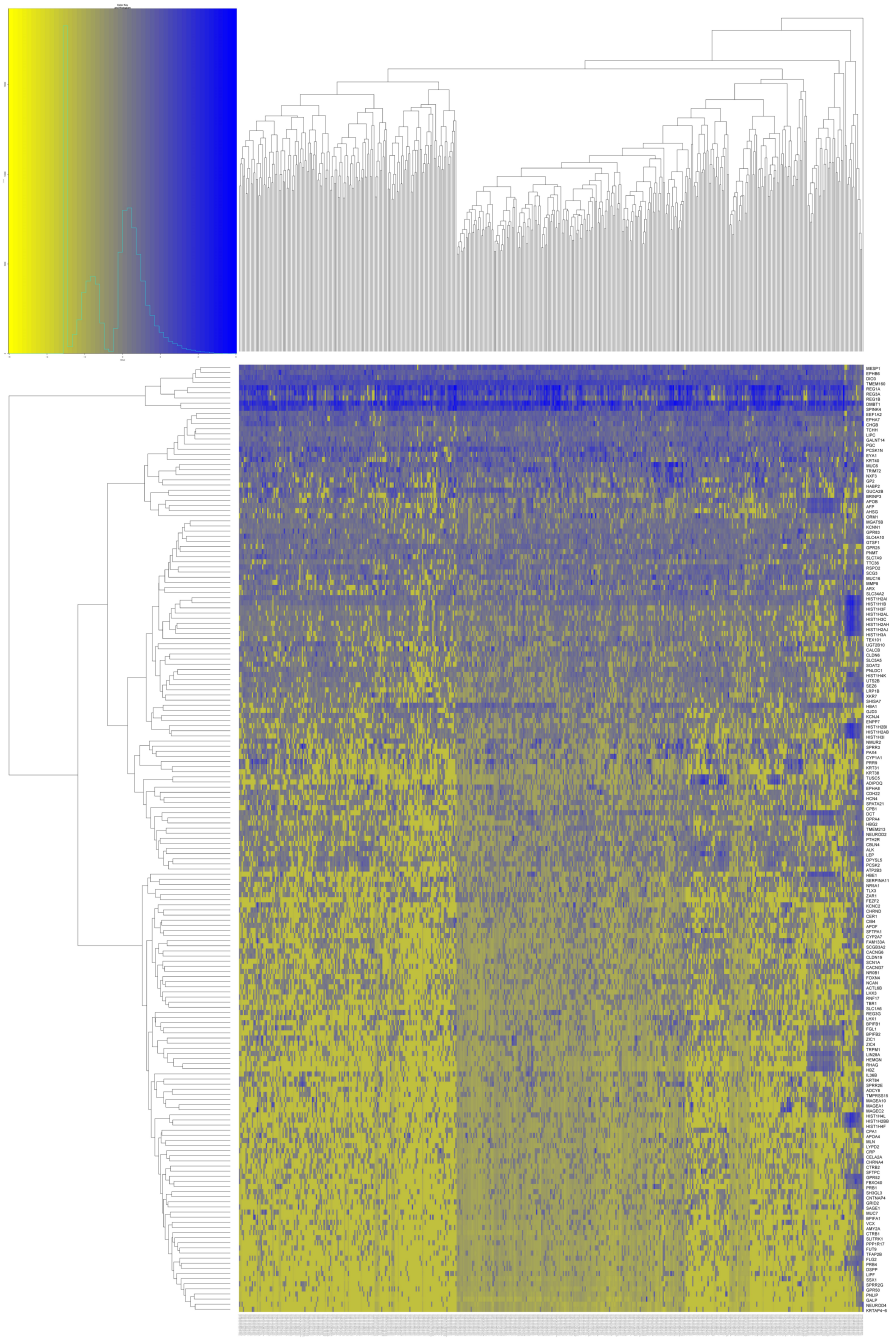
Heatmap of the DEGs related to IL20RA expression of CRC patients in TCGA. Blue suggested positively related genes and yellow suggest negatively related genes.

We separated 488 CRC patients into two groups according to the median of IL20RA expression (see Table 4 for details). We screened out IL20RA-related DEGs between patients with high and low expression of IL20RA using the R package “edgeR”. A total of 203 genes were obtained, including 55 upregulation genes and 148 downregulation genes (Table S2 for detailed data). Figure 5 shows the heat map of IL20RA-related DEGs.

**Table 4:** Information about cases of CRC in TCGA.

**Characteristics**	**Number (%)**
Total cases	488
Average age (mean ± SD) **Gender**	66.86 ± 12.57
Male	261 (53.48%)
female	227 (46.52%)
**Race category**	
Asian	9 (1.84%)
White Black Others **Cancer type** COAD READ **Disease stage** Stage I Stage II Stage III Stage IV not reported **IL20RA express** IL20RA express (median (interquartile Range) IL20RA expression (High) IL20RA expression (Low)	229 (46.93%) 57 (11.68%) 193(39.55%) 399 (81.76%) 89 (18.24%) 88 (18.03%) 186 (38.11%) 128 (26.23%) 71 (14.55%) 15 (3.08%) 996.5 (712.2–1347) 244 (50.00%) 244 (50.00%)

### Gene Ontology (GO) and KEGG pathway analysis of IL20RA-related DEGs

**Figure 6:**
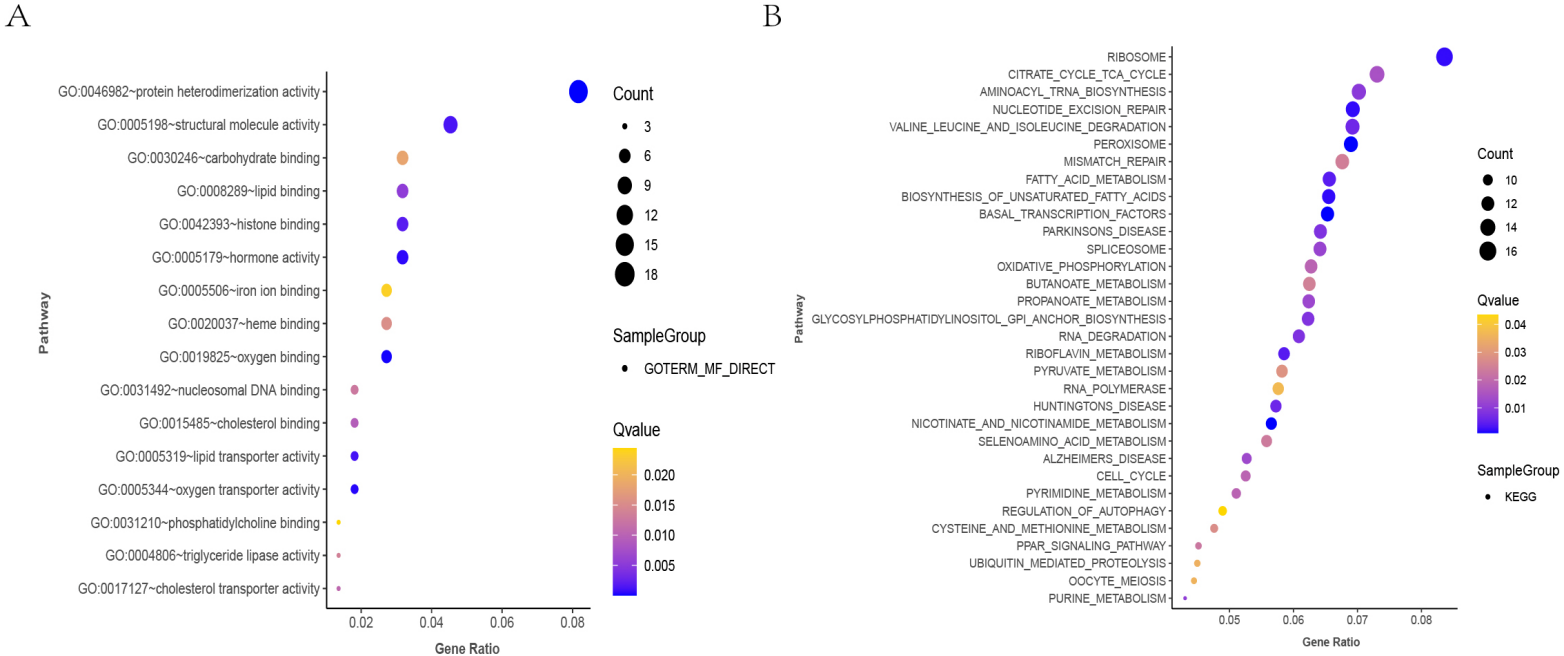
GO and KEGG analysis of DEGs associated with differential expression of IL20RA. (A) Bubble chart of functional enrichment analysis (GO analysis) of related genes that are highly expressed in IL20RA in CRC. The gene ratio is specified as the *x*-axis and the description of the path to the *y*-axis. The area of the displayed graphic is proportional to the number of genes assigned to the term and the color corresponds to the P value. (B) Bubble diagram of pathway enrichment analysis (KEGG analysis) of IL20RA overexpressing related DEGs in CRC. The gene ratio is specified as the *x*-axis and the description of the path to the *y*-axis. The area of the displayed graphic is proportional to the number of genes assigned to the term and the color corresponds to the *P* value.

GO term analysis was used to perform functional analysis of IL20RA-related DEGs, which were associated with protein heterodimerization activity, oxygen binding, oxygen transporter activity, hormone activity, and lipid transporter activity, etc (Fig. 6A). KEGG analysis was for potential pathway exploring which demonstrated the IL20RA-related DEGs were mainly enriched in peroxidase, nucleotide-stimulated repair, fatty acid metabolism, basic transcription factors, and RNA degradation (Fig. 6B).

### PPI network of IL20RA-related DEGs

**Figure 7:**
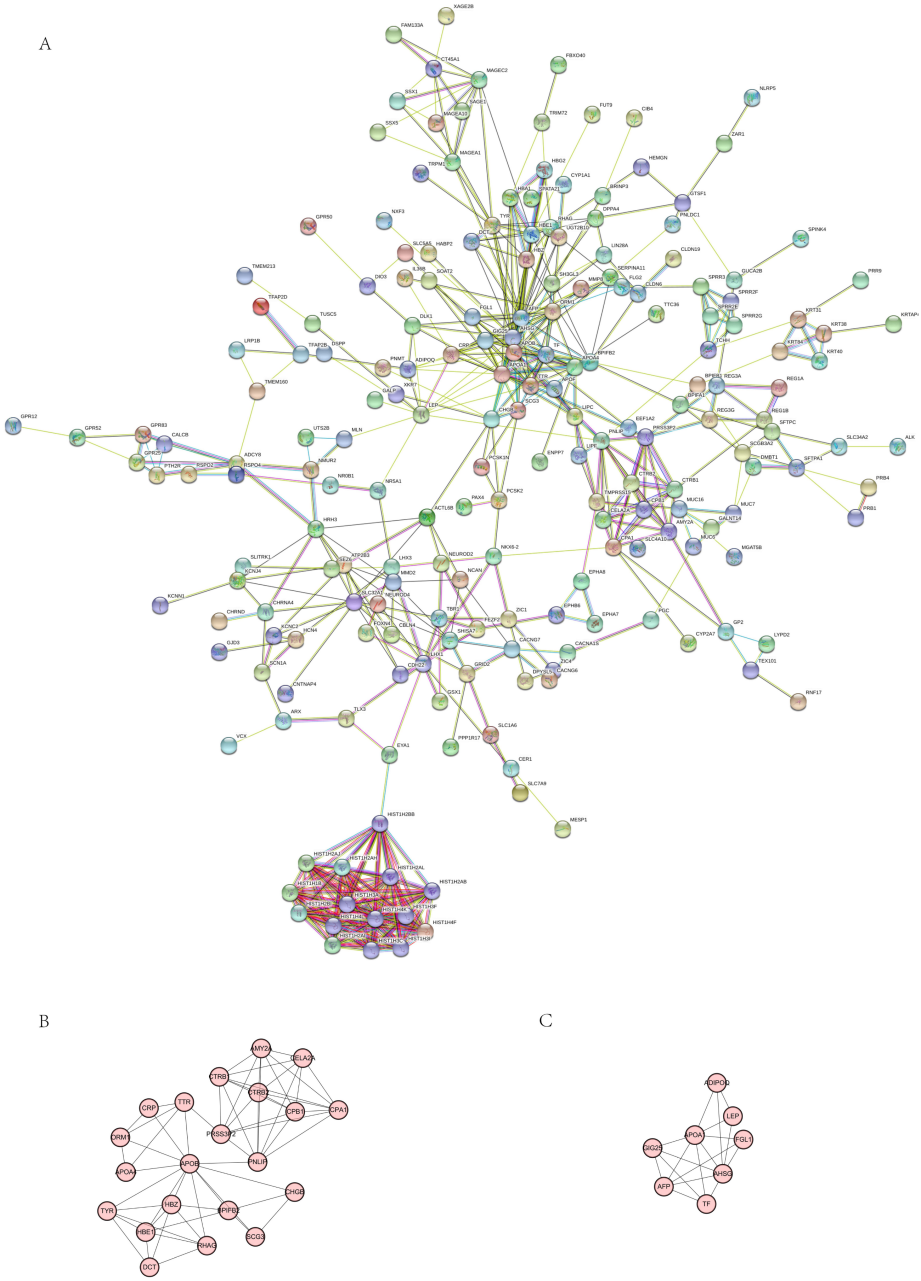
The PPI Network of DEGs related to IL20RA high expression. (A) Protein-protein interaction (PPI) network of the DEGs to IL20RA high expression in CRC. (B) The stable PPI network of core gene APOB. (C) The stable PPI network of core gene APOA1.

PPI network constructed by STRING database was to discover the potential gene-gene interactions associated with IL20RA-related DEGs in CRC (Fig. 7A). The top 5 hub genes (APOB, AHSG, AFP, APOA1, and HIST1H2BB) were identified in Table 5. MOCE was applied to perform K-score analysis to screen out a stable network structure. APOB and APOA1 were found to be the most significant core genes according to the importance of key genes and the network structure above, which were mainly related to lipoprotein synthesis, and participate in the metabolism and transportation of fatty acids (Figs. 7B, 7C).

## Discussion

In this study, we found that IL20RA protein expression was increased in CRC, which was consistent with the IL20RA mRNA expression in the Oncomine database. In subgroup analysis, the high expression of IL20RA protein was associated with CRC tumor size, lymph node metastasis, poor TNM stage, and shorter survival time, but the expression of IL20RA did not correlate with that of Ki-67, P53, EGFR. In addition, the ROC result suggested the IL20RA might perform well in CRC prognosis. We used bioinformatics to conduct a comprehensive analysis of genomics in CRC tissue based on the expression of IL20RA. In CRC, a total of 203 DEGs were identified between the IL20RA high expression group and the low expression group. Further gene enrichment and PPI network analysis were successively identified certain functions, pathways, and core genes matched with IL20RA expression in CRC. Thus, it was suggested that the expression of IL20RA might play a vital role in the development of CRC.

Previous research found that IL20RA protein was upregulated in non-small cell lung cancer (Baird, Gray & O’Byrne, 2011), but no reports have been published about IL20RA protein expression in CRC. In this study, we first found that IL20RA protein was upregulated in CRC compared with the level in adjacent normal tissues, which was not influenced by sex, age, or tumor site. Oncomine database analysis also showed a significant increase in IL20RA mRNA expression in CRC tissues compared with the level in normal tissues. Some underlying mechanisms might influence IL20RA expression, such as promoter methylation. Wigle et al. showed that IL20RA was downregulated by the hypermethylation of DNA CpG islands (Wigle et al., 2002). In addition, Liu et al. reported that IL20RA was hypomethylated and highly expressed in CRC (Liu et al., 2017). These results suggested that IL20RA might serve as a tumor biomarker for predicting CRC.

**Table 5:** The hub genes related to IL20RA overexpression in CRC patients.

**Gene Name**	**Degree**	**Betweenness**	**Closeness**
APOB	26	7141.337763	0.00144928
AHSG	26	4684.386448	0.00140647
AFP	23	4295.230313	0.00136426
APOA1	21	1942.204088	0.00138504
HIST1H2BB	15	5348.153846	8.12E−04

To clarify the clinical significance of IL20RA expression, we further explored its relationship with CRC clinicopathological parameters and prognosis. In the analysis of clinical characteristics, we found that higher expression of IL20RA protein was associated with poor biological behavior, such as larger tumor size, lymph node metastasis, and poor TNM stage. These results indicated that high expression of IL20RA might contribute to an aggressive phenotype of CRC. We also performed a comparison of survival in the high- and low-IL20RA-expression groups. The results showed that CRC patients with the high IL20RA expression exhibited significantly shorter survival than the low ones. In contrast to our results, a previous study showed that the low expression of IL20RA was related to the poor prognosis in non-small cell lung cancer (Wigle et al., 2002). Furthermore, by multivariate Cox regression analysis, we revealed that the overall survival of CRC was related not only to the expression of IL20RA protein but also to the degree of tumor differentiation and TNM stage. Some literature also demonstrated the poor behaviors that IL20RA was correlated with were the important factors affecting patients’ tumor-related death (Vatandoust, Price & Karapetis, 2015). Consequently, we believed that IL20RA could be used as a reference for predicting the prognosis of CRC patients. However, there is a need for further research and clarification regarding the specific mechanism by which IL20RA functions.

Based on the expression characteristics, the association with the clinicopathological parameters, and the prognostic value of IL20RA in CRC, we further explored the IL20RA expression-related genes and interaction networks by bioinformatic analysis. Some IL20RA-related DEGs were newly identified in our study. GO analysis suggested the IL20RA-related DEGs were mainly associated with oxygen transporter activity, oxygen binding, and protein heterodimerization activity while the KEGG analysis showed participation in peroxidase, nucleotide stimulant repair, and fatty acid metabolism, etc. The hypoxic microenvironment was significant for the rapid growth of solid tumors and contributing to the local invasion, lymph node metastasis(Cairns, Harris & Mak, 2011), and resistance to radio-chemotherapy (De la Puente et al., 2016) of solid tumors. Therefore, we proposed that the high protein expression of IL20RA was associated with oxygen binding and oxygen transporter activity, which might disturb the hypoxic microenvironment of tumor tissues in CRC. In addition, high expression of IL20RA protein was correlated with hormone activity as well. Tumor formation and development often built a strong connection with abnormal hormone and receptor levels. For example, estrogen and progesterone receptors were known to play an important role in the development of breast cancer. Meanwhile, growth hormone receptors were highly expressed in CRC which was closely linked to worse prognosis (Yang, Huang & Wang, 2010). However, the mechanisms of IL20RA affecting CRC through hormone regulation still need further exploration. Furthermore, APOB and APOA1 were found to be most associated with the high protein expression of IL20RA among all IL20RA-related DEGs through PPI network analysis. They were mainly related to lipoprotein synthesis and participating in metabolism and transportation of fatty acids. APOB, previously identified as a candidate gene related to lipid abnormality, was proved significant in the incidence of CRC and lung cancer (Borgquist et al., 2016) while APOA1 was correlated with the incidence of breast cancer (Borgquist et al., 2016). The results implied the high expression of IL20RA might be vital in lipoprotein synthesis and the metabolism and transportation of fatty acids as well as other studies have already shown the close contact between lipid metabolism and the occurrence, development, and metastasis of CRC (Peng et al., 2018). Evidence also revealed a causal relationship among hypercholesterolemia, hypertriglyceridemia, and CRC (Tie et al., 2017). It has been asserted that a high-fat diet could cause the formation of abnormal crypt foci, adenoma, and adenocarcinoma by promoting inflammation, resulting in metabolic abnormalities, and affecting cell cycle (Qi et al., 2015). In future, the relationship between IL20RA and fatty acid metabolism in CRC may lead to more novel findings.

In conclusion, in our study, IL20RA protein expression, as well as mRNA expression, was upregulated in CRC tissues. This suggested that IL20RA might act as a new biomarker of CRC. Furthermore, high IL20RA protein expression might predict an aggressive CRC phenotype and poor prognosis. We also speculated that IL20RA is involved in the development and progression of CRC by affecting fatty acid metabolism, oxygen binding, oxygen transport, and hormone activity, among others. These characteristics of IL20RA expression in tumors suggest that it could act as an auxiliary index for evaluating the risk and prognosis of CRC.

## Conclusions

IL20RA might have potential to act as a biomarker for CRC. The upregulation of IL20RA might contribute to an aggressive phenotype of CRC. Specifically, IL20RA might be involved in the development and progression of CRC by affecting fatty acid metabolism, oxygen binding, oxygen transport, and hormone activity.

##  Supplemental Information

10.7717/peerj.12467/supp-1 Supplemental Information 1 Supplemental Tables

10.7717/peerj.12467/supp-2 Supplemental Information 2 Raw data for Figs. 1 and 2

10.7717/peerj.12467/supp-3 Supplemental Information 3 Raw data

## Additional Information and Declarations

### Data Availability

The following information was supplied regarding data availability: Raw data are available in the Supplemental Files.
