# Supplementary material for: Interleukin 20 receptor A expression in colorectal cancer and its clinical significance
Source: PeerJ. 2021 Nov 16;9:e12467. doi: 10.7717/peerj.12467 (PMC8603834; doi:10.7717/peerj.12467)
Supplement: Supplemental Information 1 [file peerj-09-12467-s001.docx]

**Supplemental Table 1: Correlation of IL20RA expression with commonly used biomarkers (n=120)**

| IL20RA | KI67(n) | | | |  | P53(n) | | | |  | EGFR(n) | | | |
| --- | --- | --- | --- | --- | --- | --- | --- | --- | --- | --- | --- | --- | --- | --- |
|  | - | + | ++ | +++ |  | - | + | ++ | +++ |  | - | + | ++ | +++ |
| 0 | 0 | 0 | 0 | 13 |  | 2 | 2 | 0 | 9 |  | 12 | 0 | 0 | 1 |
| 1 | 2 | 2 | 6 | 54 |  | 24 | 3 | 5 | 32 |  | 49 | 3 | 5 | 7 |
| 2 | 2 | 0 | 4 | 24 |  | 12 | 4 | 1 | 13 |  | 22 | 2 | 1 | 5 |
| 3 | 0 | 0 | 1 | 12 |  | 4 | 0 | 1 | 8 |  | 11 | 1 | 0 | 1 |
| R value | 0.004 | | | |  | -0.037 | | | |  | 0.027 | | | |
| *p*-value | 0.967 | | | |  | 0.685 | | | |  | 0.774 | | | |

**Supplemental Table 2: IL20RA-related DEGs obtained from TCGA database**

| **ID** | **logFC logCPM PValue** | **FDR** |
| --- | --- | --- |

| DIO3 | 2.358111 | 14.37045025 | 6.10E-37 | 1.20E-34 |
| --- | --- | --- | --- | --- |
| LIPC | 2.72714 | 11.82337095 | 1.38E-33 | 1.35E-31 |
| EYA1 | 2.657628 | 13.0838713 | 2.91E-29 | 1.90E-27 |
| SLC4A10 | 2.210633 | 10.81049921 | 8.54E-27 | 3.70E-25 |
| MMP8 | 3.099581 | 11.94943811 | 9.43E-27 | 3.70E-25 |
| CPA1 | 4.326895 | 9.01714932 | 2.14E-26 | 7.00E-25 |
| SFTPC | 5.650814 | 10.72560797 | 7.86E-25 | 2.20E-23 |
| HIST1H3F | 3.241218 | 12.09683904 | 3.43E-24 | 7.69E-23 |
| SFTPA1 | 4.218002 | 10.10999597 | 3.53E-24 | 7.69E-23 |
| UGT2B10 | 2.543624 | 10.54015741 | 1.72E-22 | 3.37E-21 |
| GPR83 | 2.360354 | 11.22487937 | 3.05E-22 | 4.99E-21 |
| HBA1 | -1.94058 | 10.07243119 | 3.06E-22 | 4.99E-21 |
| FAM133A | 3.026942 | 8.679607169 | 7.13E-22 | 1.07E-20 |
| KCNC2 | 4.229114 | 10.24434422 | 1.72E-21 | 2.41E-20 |
| FEZF2 | -2.30631 | 7.764751694 | 2.83E-21 | 3.70E-20 |
| TCHH | 1.996332 | 11.48447277 | 5.32E-21 | 6.52E-20 |
| REG1B | -2.82228 | 17.31492628 | 1.06E-18 | 1.22E-17 |
| REG1A | -2.41269 | 19.7364442 | 1.29E-18 | 1.41E-17 |
| TFAP2B | -3.47202 | 7.796352366 | 4.06E-18 | 4.19E-17 |
| CACNG6 | 2.994089 | 8.70939875 | 4.39E-18 | 4.30E-17 |
| CTRB2 | 2.825355 | 7.891002059 | 4.74E-17 | 4.42E-16 |
| HBZ | -3.37094 | 7.849002451 | 8.49E-17 | 7.36E-16 |
| CTRB1 | 3.388357 | 7.732066337 | 8.63E-17 | 7.36E-16 |
| HIST1H1B | 2.612736 | 13.95422157 | 9.05E-17 | 7.39E-16 |
| GALP | 4.579872 | 7.869089342 | 1.32E-16 | 1.03E-15 |
| CELA2A | 2.547199 | 7.857076093 | 1.60E-16 | 1.14E-15 |
| AHSG | -1.9616 | 10.06727922 | 1.61E-16 | 1.14E-15 |
| KRT84 | 3.162354 | 9.291607454 | 1.63E-16 | 1.14E-15 |
| GP2 | -2.51556 | 13.36480559 | 1.87E-16 | 1.27E-15 |
| HIST1H2AI | 2.037706 | 12.16221821 | 1.99E-16 | 1.30E-15 |
| HIST1H4K | 1.71562 | 9.271955564 | 3.66E-16 | 2.31E-15 |
| HIST1H2AJ | 2.509138 | 11.48664779 | 1.37E-15 | 8.42E-15 |
| LIN28A | -2.18776 | 8.102227054 | 2.71E-15 | 1.61E-14 |
| ORM1 | 2.75199 | 13.44873114 | 3.54E-15 | 2.04E-14 |
| HIST1H3C | 2.403165 | 11.70117382 | 1.94E-14 | 1.09E-13 |
| NMUR2 | -2.39442 | 11.04181653 | 3.86E-14 | 2.10E-13 |
| LRP1B | -1.88395 | 9.213591213 | 4.08E-14 | 2.16E-13 |
| ADCY8 | -2.15535 | 7.677501861 | 4.67E-14 | 2.39E-13 |
| HIST1H2AL | 2.243411 | 11.04785607 | 4.75E-14 | 2.39E-13 |
| HIST1H3A | 2.126357 | 10.30450532 | 1.38E-13 | 6.76E-13 |
| PNLIP | 4.732191 | 8.775723399 | 1.80E-13 | 8.61E-13 |
| HIST1H2BI | 2.68286 | 11.59988536 | 1.30E-12 | 6.09E-12 |
| KRT31 | 2.660118 | 10.09258395 | 1.90E-12 | 8.66E-12 |
| REG3A | -1.97364 | 18.29891835 | 6.82E-12 | 3.04E-11 |
| NR5A1 | -1.69109 | 8.388354978 | 1.65E-11 | 7.19E-11 |
| PTH2R | 2.169045 | 9.953918059 | 1.72E-11 | 7.32E-11 |
| HIST1H3I | 2.229697 | 10.67985911 | 2.01E-11 | 8.37E-11 |
| CALCB | -2.38649 | 12.970321 | 3.95E-11 | 1.61E-10 |
| TMEM160 | -0.7493 | 14.84511316 | 4.94E-11 | 1.98E-10 |
| HIST1H2AB | 2.143746 | 10.74413105 | 5.13E-11 | 2.01E-10 |
| LEP | 1.786111 | 9.689258062 | 6.46E-11 | 2.48E-10 |
| HIST1H2AH | 1.951198 | 11.50436131 | 7.26E-11 | 2.74E-10 |
| RHAG | -2.11076 | 7.52775229 | 8.32E-11 | 3.08E-10 |
| CRP | 2.434006 | 8.217810778 | 8.97E-11 | 3.26E-10 |
| EPHB6 | 1.390774 | 14.40372445 | 1.90E-10 | 6.79E-10 |
| NR0B1 | -1.62902 | 7.704031418 | 2.08E-10 | 7.27E-10 |
| HIST1H4F | 3.092615 | 9.341611593 | 2.18E-10 | 7.51E-10 |
| SPRR2E | -3.10019 | 10.9467671 | 4.33E-10 | 1.46E-09 |
| MLN | 2.588991 | 9.076344587 | 7.46E-10 | 2.48E-09 |
| PRR9 | 2.233777 | 11.42713378 | 8.65E-10 | 2.83E-09 |
| PNMT | -1.11079 | 10.09537967 | 1.48E-09 | 4.76E-09 |
| LIPF | -2.72844 | 8.13084908 | 1.94E-09 | 6.13E-09 |
| TMPRSS15 | -1.76763 | 7.699596917 | 4.34E-09 | 1.35E-08 |
| HIST1H4L | 2.748825 | 10.73437433 | 6.39E-09 | 1.96E-08 |
| PGC | -1.17381 | 12.28937388 | 7.41E-09 | 2.23E-08 |
| NEUROD4 | -2.03868 | 7.078318389 | 7.93E-09 | 2.36E-08 |
| UTS2B | -1.23373 | 9.051265355 | 8.94E-09 | 2.61E-08 |
| SSX1 | 2.405494 | 7.467872481 | 9.27E-09 | 2.67E-08 |
| MUC16 | -1.47413 | 12.08628009 | 1.09E-08 | 3.08E-08 |
| GJD3 | -1.01246 | 8.307860681 | 1.74E-08 | 4.88E-08 |
| MUC7 | -2.24569 | 8.192090627 | 1.83E-08 | 5.02E-08 |
| CPB1 | 1.763862 | 9.452125735 | 1.85E-08 | 5.02E-08 |
| EEF1A2 | -0.96229 | 12.06154587 | 1.93E-08 | 5.19E-08 |
| HIST1H2BB | 2.938109 | 10.86261632 | 2.20E-08 | 5.82E-08 |
| EPHA7 | -1.06528 | 12.29209868 | 2.49E-08 | 6.50E-08 |
| BPIFB2 | -2.00356 | 8.772953248 | 2.55E-08 | 6.59E-08 |
| AFP | -1.12445 | 9.701820829 | 3.51E-08 | 8.94E-08 |
| MAGEC2 | 3.083446 | 10.49106537 | 3.88E-08 | 9.75E-08 |
| FUT9 | -1.91979 | 7.475763824 | 4.22E-08 | 1.05E-07 |
| CHRND | -1.86709 | 9.028509644 | 5.34E-08 | 1.31E-07 |
| KRT38 | 1.742563 | 8.239419209 | 6.57E-08 | 1.59E-07 |
| NXF3 | -1.32661 | 12.28400988 | 2.19E-07 | 5.22E-07 |
| AMY2A | 1.77971 | 7.551231309 | 5.06E-07 | 1.19E-06 |
| CACNG7 | -1.13561 | 7.687850162 | 5.31E-07 | 1.24E-06 |
| KRTAP4-6 | -1.7861 | 7.065549228 | 7.59E-07 | 1.75E-06 |
| IL36B | -1.47952 | 8.480492645 | 9.87E-07 | 2.25E-06 |
| SLC34A2 | -1.22172 | 10.70002379 | 1.63E-06 | 3.68E-06 |
| REG3G | -1.64634 | 9.12739705 | 1.73E-06 | 3.81E-06 |
| APOB | -1.26325 | 11.40418625 | 1.73E-06 | 3.81E-06 |
| GALNT14 | -0.89245 | 11.26834913 | 1.86E-06 | 4.04E-06 |
| PPP1R17 | -1.52162 | 7.339111367 | 2.00E-06 | 4.31E-06 |
| MUC6 | -1.42479 | 15.49623216 | 2.50E-06 | 5.33E-06 |
| HBE1 | -1.76074 | 9.773336323 | 2.90E-06 | 6.12E-06 |
| MGAT5B | -0.6748 | 9.142226183 | 4.56E-06 | 9.52E-06 |
| PRB1 | -1.9127 | 8.096448012 | 5.95E-06 | 1.23E-05 |
| ENPP7 | -0.91826 | 8.303943488 | 7.96E-06 | 1.62E-05 |
| HBG2 | -1.00983 | 8.042970579 | 9.35E-06 | 1.89E-05 |
| ARX | -1.07038 | 10.55217767 | 9.44E-06 | 1.89E-05 |
| MAGEA10 | -1.8582 | 9.346622554 | 1.54E-05 | 3.05E-05 |
| HCN4 | -0.76342 | 8.030283434 | 3.29E-05 | 6.45E-05 |
| APOA4 | -1.79976 | 9.206544671 | 3.45E-05 | 6.69E-05 |
| ZIC1 | -1.3132 | 8.940595502 | 3.55E-05 | 6.83E-05 |
| TRIM72 | -1.00603 | 13.19749868 | 4.58E-05 | 8.72E-05 |
| TEX101 | -0.94199 | 9.49994977 | 5.21E-05 | 9.82E-05 |
| TUSC5 | 1.481267 | 10.1793023 | 5.41E-05 | 0.000101 |
| CIB4 | -1.01097 | 8.230418945 | 7.43E-05 | 0.000137 |
| TTC36 | -0.54931 | 8.608132865 | 9.01E-05 | 0.000165 |
| HABP2 | -1.06878 | 12.33396672 | 0.000117 | 0.000212 |
| CBLN4 | -1.05189 | 8.997772274 | 0.000121 | 0.000218 |
| DSPP | -1.35291 | 7.291144594 | 0.00013 | 0.000232 |
| VCX | 1.31917 | 7.490464909 | 0.000164 | 0.000285 |
| BRINP3 | -1.05797 | 11.22053066 | 0.000164 | 0.000285 |
| GPR50 | -1.1989 | 7.088623023 | 0.000164 | 0.000285 |
| SLC5A5 | -0.72373 | 8.523623109 | 0.000168 | 0.000288 |
| FBXO40 | -1.57393 | 8.344547076 | 0.000173 | 0.000295 |
| DPPA4 | -0.7287 | 8.000175499 | 0.000193 | 0.000326 |
| MAGEA1 | -1.9847 | 11.00868172 | 0.000241 | 0.000404 |
| TMEM213 | -0.87909 | 8.924353125 | 0.00028 | 0.000465 |
| RSPO2 | -0.67592 | 10.3444021 | 0.000288 | 0.000475 |
| CDH22 | 1.118051 | 9.927383113 | 0.000415 | 0.000677 |
| MESP1 | -0.48252 | 12.74063953 | 0.000475 | 0.000763 |
| NCAN | -0.75641 | 7.874792637 | 0.000475 | 0.000763 |
| SLC7A9 | -0.7291 | 11.12940577 | 0.000506 | 0.000806 |
| LHX1 | -1.00887 | 8.090073657 | 0.000781 | 0.001234 |
| KRT40 | -0.93591 | 13.86142911 | 0.000877 | 0.001369 |
| DPYSL5 | -0.6763 | 8.670851098 | 0.00088 | 0.001369 |
| CHRNA4 | -0.93533 | 7.571060377 | 0.001008 | 0.001556 |
| ZIC4 | -1.07419 | 7.74643139 | 0.001215 | 0.00186 |
| SOAT2 | -0.75338 | 9.315899289 | 0.001445 | 0.002196 |
| PCSK1N | -0.73227 | 13.78207722 | 0.001611 | 0.002428 |
| GPR25 | -0.4765 | 9.668025898 | 0.001645 | 0.002461 |
| ALK | -0.75968 | 9.026038779 | 0.001657 | 0.002461 |
| SCG3 | -1.83836 | 10.75743151 | 0.001812 | 0.00267 |
| GRID2 | -0.88641 | 7.427396167 | 0.001982 | 0.002899 |
| SHISA7 | -0.58523 | 7.937082839 | 0.002118 | 0.003075 |
| HEMGN | -0.71963 | 7.371845639 | 0.002212 | 0.003188 |
| KCNN1 | -0.38513 | 8.818509297 | 0.002262 | 0.003236 |
| FLG2 | -1.00954 | 7.386463693 | 0.002799 | 0.003975 |
| ADIPOQ | 1.354791 | 11.94139308 | 0.00309 | 0.004357 |
| SERPINA11 | -0.82362 | 8.842480839 | 0.003193 | 0.00447 |
| PRB4 | -1.01516 | 7.384515336 | 0.004416 | 0.006122 |
| CYP1A1 | -0.9963 | 10.77587283 | 0.004435 | 0.006122 |
| SLITRK1 | -0.81577 | 7.167653438 | 0.004467 | 0.006123 |
| TBR1 | -0.71174 | 7.388722801 | 0.004511 | 0.00614 |
| TRPM1 | -0.73649 | 7.644466767 | 0.008315 | 0.01124 |
| PAX4 | -0.78677 | 9.650989852 | 0.008714 | 0.011698 |
| LHX3 | -0.74623 | 7.844896035 | 0.009658 | 0.012878 |
| TLX3 | -0.84504 | 8.731911759 | 0.010319 | 0.013666 |
| CHGB | 0.582729 | 13.27598849 | 0.012107 | 0.015926 |
| SLC1A6 | -0.60564 | 7.285259488 | 0.017943 | 0.023445 |
| DCT | -0.59188 | 9.972474558 | 0.018185 | 0.023604 |
| SPRR3 | -0.90162 | 12.17988844 | 0.019705 | 0.025409 |
| CLDN19 | -0.56889 | 7.607215166 | 0.021893 | 0.028045 |
| ZAR1 | -0.46026 | 7.920283106 | 0.024884 | 0.031671 |
| NEUROD2 | -0.46331 | 8.199842489 | 0.03347 | 0.042323 |
| CYP2A7 | -0.48453 | 7.476814554 | 0.035201 | 0.044013 |
| DMBT1 | -0.44377 | 19.55714216 | 0.035255 | 0.044013 |
| PNLDC1 | -0.37477 | 9.13145801 | 2.25E-14 | 5.27E-13 |
| ATP2B3 | -0.43141 | 7.847963168 | 1.32E-11 | 1.98E-10 |
| APOF | -0.46191 | 7.7410233 | 6.14E-11 | 8.24E-10 |
| PCSK2 | -0.45984 | 8.770363655 | 2.54E-09 | 2.49E-08 |
| CLDN6 | -0.43236 | 9.503939999 | 1.28E-19 | 6.33E-18 |
| SPRR2G | 0.884371 | 7.561305746 | 1.53E-07 | 1.03E-06 |
| FOXN4 | -0.48418 | 7.751172621 | 2.32E-10 | 2.78E-09 |
| SPINK4 | -0.41201 | 17.4049961 | 1.4E-07 | 9.57E-07 |
| KCNJ4 | -0.37715 | 8.656724437 | 7.85E-11 | 1.04E-09 |
| SCGB3A2 | -0.5414 | 7.965528029 | 1.93E-06 | 1.02E-05 |
| ACTL6B | -0.43944 | 7.548411603 | 1.97E-10 | 2.41E-09 |
| BPIFB1 | -0.67203 | 9.203038297 | 2.64E-05 | 0.000107 |
| CNTNAP4 | -0.50364 | 7.358132308 | 6.23E-11 | 8.35E-10 |
| GPR52 | -0.56975 | 7.971702307 | 6.74E-06 | 3.14E-05 |
| SCN1A | -0.49741 | 7.810498751 | 8.01E-09 | 7.07E-08 |
| LYPD2 | -0.49133 | 7.466408751 | 7.74E-06 | 3.55E-05 |
| SH3GL3 | -0.44975 | 7.327306481 | 2.03E-08 | 1.66E-07 |
| CER1 | -0.49896 | 8.219130732 | 3.54E-15 | 9.3E-14 |
| EPHA8 | -0.44978 | 9.756268095 | 1.38E-08 | 1.16E-07 |
| RNF17 | -0.3862 | 7.646467465 | 3.83E-10 | 4.39E-09 |
| FGL1 | -0.39867 | 8.492529502 | 4.36E-08 | 3.34E-07 |
| BPIFA1 | -0.67477 | 8.569568982 | 2.72E-15 | 7.25E-14 |
| SEZ6 | -0.29027 | 9.12478054 | 5.3E-14 | 1.16E-12 |
| GTSF1 | -0.2889 | 10.54382118 | 3.61E-10 | 4.14E-09 |
| SPATA21 | -0.27846 | 8.143523754 | 2.21E-09 | 2.19E-08 |
| XKR7 | 0.35618 | 11.25250697 | 4.01E-08 | 3.09E-07 |
| SAGE1 | -0.39102 | 8.227663723 | 6.46E-06 | 3.03E-05 |
| GUCA2B | -0.20819 | 11.51698779 | 1.79E-07 | 1.19E-06 |
| TTR | -0.15535 | 10.84393476 | 6.04E-11 | 8.11E-10 |
| TF | -0.12077 | 10.92110427 | 3.01E-13 | 5.83E-12 |
| SPRR2F | -0.30122 | 9.663803525 | 5.81E-16 | 1.7E-14 |
| DLK1 | -0.1949 | 8.761956103 | 9.82E-06 | 4.42E-05 |
| CT45A1 | -0.22723 | 7.880333536 | 5.71E-18 | 2.25E-16 |
| TYR | -1.9012 | 8.145267434 | 2.63E-07 | 1.69E-06 |
| APOA1 | -0.0697 | 10.53653559 | 1.97E-08 | 1.61E-07 |
| HRH3 | -0.12239 | 7.901836974 | 5.76E-07 | 3.43E-06 |
| GSX1 | -0.09017 | 7.671510924 | 2.95E-09 | 2.84E-08 |
| CACNA1S | -1.54785 | 8.175225822 | 6.06E-10 | 6.7E-09 |
| RSPO4 | -0.01854 | 10.69974582 | 2.92E-07 | 1.85E-06 |
| TFAP2D | -3.52113 | -3.686425727 | 1.20E-19 | 6.00E-18 |
| SLC32A1 | -3.21787 | -3.456048511 | 2.24E-21 | 1.38E-19 |
| SSX5 | -2.20932 | -3.380740594 | 4.68E-07 | 2.85E-06 |
| NLRP5 | -2.18965 | -3.66879639 | 1.77E-10 | 2.17E-09 |
| MMD2 | -1.84094 | -3.689494812 | 5.11E-08 | 3.84E-07 |
| NKX6-2 | -1.65353 | -3.52135067 | 7.55E-09 | 6.70E-08 |
| GPR12 | -1.60271 | -3.55503097 | 7.28E-07 | 4.24E-06 |
