# Supplementary figures and images for: Interleukin 20 receptor A expression in colorectal cancer and its clinical significance
Source: PeerJ. 2021 Nov 16;9:e12467. doi: 10.7717/peerj.12467 (PMC8603834; doi:10.7717/peerj.12467)

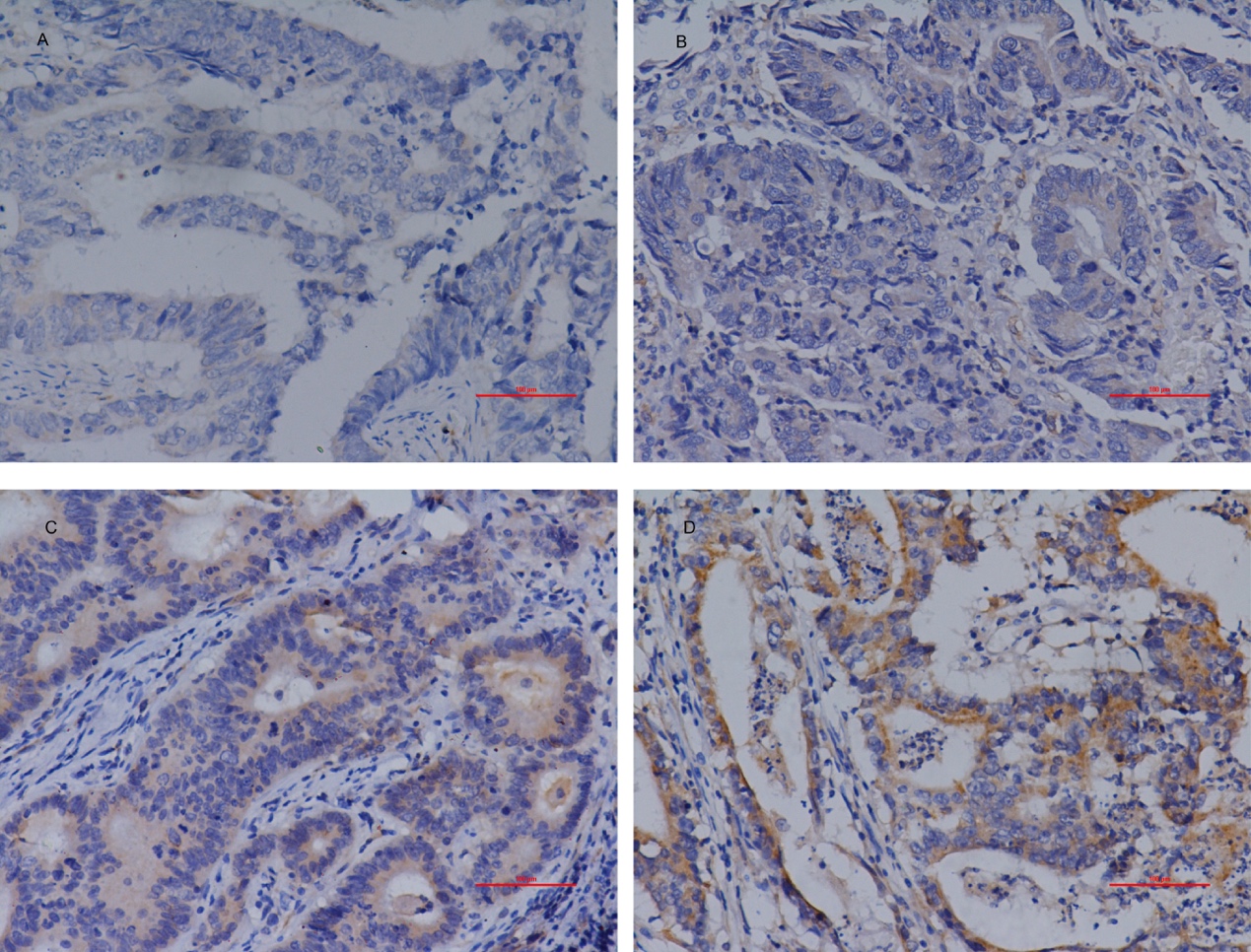

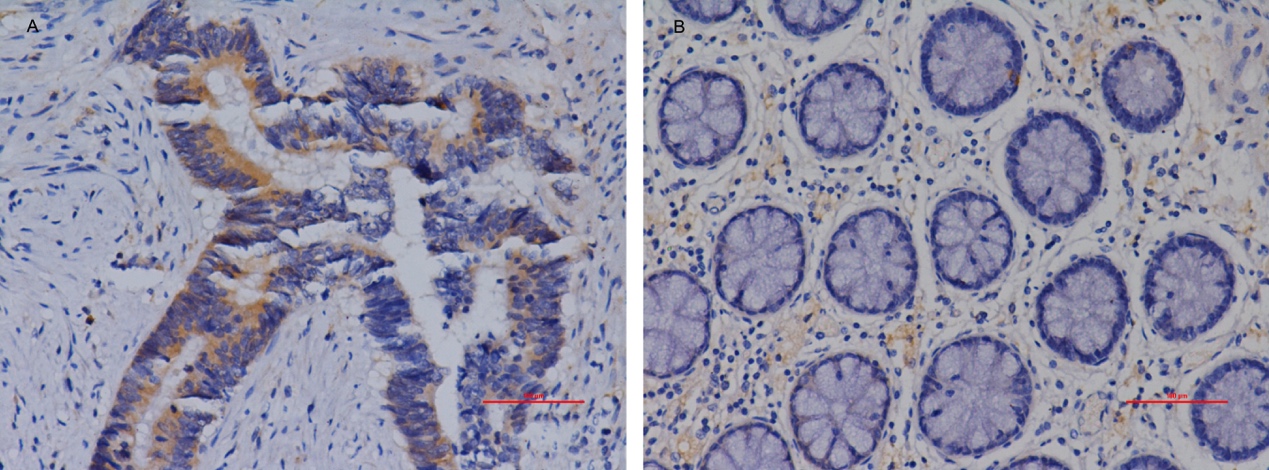


original figure 2

original figure 1

Supplement: Supplemental Information 2 [file peerj-09-12467-s002.docx]
